# Association Between Nasopharyngeal Carcinoma and Chinese Medicine Constitution: A Meta‐Analysis

**DOI:** 10.1002/cam4.71641

**Published:** 2026-03-26

**Authors:** Shun‐Qi Chen, Yan Zi, Wen‐Le Li, Yu‐Yang Cai, Geng‐Shuo Miao, Huai‐Yu Wang, Ming‐Hua Bai, Ji Wang

**Affiliations:** ^1^ Institute of Basic Theory for Chinese Medicine, China Academy of Chinese Medical Sciences Beijing China; ^2^ National Institute of Traditional Chinese Medicine (TCM) Constitution and Preventive Medicine, Beijing University of Chinese Medicine Beijing China; ^3^ College of Traditional Chinese Medicine Beijing University of Chinese Medicine Beijing China

**Keywords:** body constitution, Chinese medicine constitution, Epstein–Barr virus, meta analysis, nasopharyngeal carcinoma, review

## Abstract

**Objective:**

To explore the distribution of Chinese medicine constitution (CMC) types across the spectrum of health states related to nasopharyngeal carcinoma (NPC) and provide evidence‐based information for the prevention and treatment of NPC at different stages of the disease.

**Methods:**

PubMed, Embase, Web of Science, and three major Chinese databases were searched to retrieve literature reporting the correlation between populations across related health states of NPC and CMC, using the same standardized classification since 2009. Three authors independently screened and evaluated the quality of the methodology. The main outcomes were the single proportion and the odds ratio (OR) of each constitution type across related health states of NPC, and the effect sizes were expressed as proportions or as ORs with 95% confidence intervals (CI). Sensitivity and subgroup analyses were performed to determine the sources of heterogeneity.

**Results:**

Data of 1174 patients from 11 different studies were included in the present study. Qi‐deficiency (QD), balanced, and Yang‐deficiency constitutions accounted for 25.5% (95% CI: 19.0–32.0, *p* < 0.01), 16.1% (95% CI: 18.1–26.1), and 15.4% (95% CI: 9.5–21.4), respectively. The distribution across related health states of patients with NPC varied across different stages of the disease. QDC showed a significant association with both Epstein–Barr virus infection and NPC diagnosed status in the included populations compared with that in healthy controls.

**Conclusion:**

QDC is a major factor associated with NPC across different health statuses. However, the cross‐sectional nature of the available evidence highlights the need for more high‐quality prospective cohort studies to clarify the temporal relationship and causal role of specific constitutions in NPC development.

**Abbreviations**
AHRQ: Agency for Healthcare Research and Quality
BC/BCC: balanced constitution
BS/BSC: blood‐stasis constitution
CCS: case–control study
CI: confidence interval
CMC: Chinese medicine constitution
CSS: cross‐sectional study
DH/DHC: dampness‐heat constitution
EA‐IgA: early antigen immunoglobulin A
EBV: Epstein–Barr virus
*I* ^2^
: *I*‐squared statistic
LCI: lower confidence interval
MOOSE: Meta‐analysis Of Observational Studies in Epidemiology
NOS: Newcastle–Woodward scale
NPC: nasopharyngeal carcinoma
OR: odds ratio
PD/PDC: phlegm‐dampness constitution
PRISMA: Preferred Reporting Items for Systematic Reviews and Meta‐Analyses
QD/QDC: Qi‐deficiency constitution
QS/QSC: Qi‐stagnation constitution
RCT: randomized controlled trial
SP/SPC: special inherited constitution
TCM: traditional Chinese medicine
UCI: upper confidence interval
VCA‐IgA: viral capsid antigen immunoglobulin A
YaD/YaDC: Yang‐deficiency constitution
YiD/YiDC: Yin‐deficiency constitution

## 1 Introduction

Nasopharyngeal carcinoma (NPC) is a malignant epithelial tumor that affects many people in the eastern and southeastern regions of China [1]. According to the global cancer statistics from 185 countries reported in 2020, out of the 80,008 deaths that occurred worldwide, 24,536 occurred in China [1]. In Guangdong and Guangxi provinces of China, an age‐standardized year‐of‐life loss rate of 75.9–63.9 (per 100,000 individuals) caused by NPC has been reported [2]. Patients with NPC experience headache, nose hemorrhage, or more severe symptoms [3]. Although the diagnosis and treatment strategies of NPC have improved [4], the side effects of chemoradiotherapy to the head and neck still continue to place a burden on the patients, along with the probability of relapse [5]. The clinical situation of NPC highlights the necessity and urgency of prevention in its early stages.

The etiological factors of NPC include viral infection, such as Epstein–Barr virus (EBV) infection, lifestyle, sex, and genetic factors [6], all of which are essential for NPC development. The evidence of association between NPC and the genome shown as family aggregation has also been found, where a significant correlation of EBV loads was observed between parents–offspring and sibling–sibling pairs (*p* < 0.01) but not in distant relatives [7, 8]. These factors align with the holistic perspective of traditional Chinese medicine (TCM), which emphasizes the interactions between people and the environment [9, 10].

In TCM, an individual's “constitution” (Tizhi) refers to the relatively stable morphological structure, physiological function, and psychological state formed over a lifetime under the combined influence of innate endowment and acquired factors. It determines an individual's susceptibility to certain pathogens and their tendencies toward specific disease developments. According to the national standard “Classification and Determination of Constitution in TCM” [11], there are nine basic types: one Balanced constitution and eight Unbalanced constitutions. The unbalanced types include Qi‐deficiency, Yang‐deficiency, Yin‐deficiency, Phlegm‐dampness, Dampness‐heat, Blood‐stasis, Qi‐stagnation, and Inherited Special constitution. Among these, “deficiency” (e.g., Qi, Yang, Yin deficiency) describes a state of insufficiency or hypofunction of vital substances or energy, while “stagnation” (e.g., Qi‐stagnation, Blood‐stasis) refers to impaired flow or blockage. In particular, Qi‐stagnation is commonly associated with emotional or psychological disharmony [12], whereas Blood‐stasis is often linked to circulatory disorders or chronic pain [13]. These constitutional states can exist during suboptimal health or disease. In research and clinical practice, constitution is typically assessed and classified using standardized tools like the “Constitution in Chinese Medicine Questionnaire,” which quantifies these tendencies through self‐reported symptoms and signs. This tool has been validated and adapted into multiple languages and versions for different populations, as evidenced by its translation and validation in English [14], Japanese [15] and Korean [16], as well as its specific application in elderly population studies [17].

This theory focuses on the specific status of the human body during the entire life period [18] and states that the constitution changes earlier than the course of the disease in most cases and shows specific features in different types of constitution, especially in progressive diseases [19]. Within this paradigm of “preventive treatment of disease,” the Chinese medicine constitution theory provides an individualized framework for risk identification. Epidemiological studies have reported associations between unbalanced constitutions (e.g., Blood‐stasis, Yin‐deficiency) and various diseases [13, 20]. More importantly, contemporary research has confirmed at the molecular level that an unbalanced constitution itself represents a distinct and measurable physiological state. For instance, individuals with Qi‐deficiency or dampness‐heat constitutions exhibit specific circulating miRNA expression profiles; these molecular signatures may be involved in the pathological processes of related diseases [21, 22]. Such biological evidence underscores the potential for identifying at‐risk physiological states prior to clinical disease onset.

Building upon this foundation of risk identification, the feasibility of early intervention is paramount. Recent translational research provides compelling evidence that unbalanced constitutions are modifiable targets. Specifically, randomized controlled trials have demonstrated that targeted TCM interventions can significantly improve the biomarker profiles and clinical symptom scores characteristic of constitutions such as dampness‐heat and Qi‐deficiency [23, 24]. This evidence substantiates the core premise of preventive TCM. The predictive and prognostic value of constitution for clinical outcomes is further supported by prospective cohort study designs [25].

However, when examining the specific link between TCM constitutions and NPC, the evidence remains preliminary and presents distinct challenges. Current knowledge is fragmented across different disease stages and relies on non‐uniform assessment methods [26, 27]. These factors collectively hinder the establishment of a clear, consistent, and convincing pattern of association from the existing literature. This ambiguity consequently fails to provide a sufficient basis for initiating deeper investigations, such as cohort studies or randomized controlled trials.

To address these intertwined methodological issues and move the field forward, a systematic review and meta‐analysis is critically needed. This serves not only as a critical step to obtain higher‐quality synthesized evidence, but also helps to clearly define the knowledge baseline in this field. Doing so can draw focused scholarly attention and lay a foundation, as well as offer direction, for future prospective studies designed to verify causality or evaluate preventive interventions.

## 2 Methods

In this study, we followed the Preferred Reporting Items for Systematic Reviews and Meta‐Analyses guidelines [28, 29] (File S1) and the Meta‐analysis of Observational Studies in Epidemiology reporting guidelines on JAMA [30]. This study was registered in PROSPERO (https://www.crd.york.ac.uk/PROSPERO/, ID CRD42022292870).

### 2.1 Retrieval Strategy

Two authors (Chen and Li) performed literature searches during the preparation period and conducted independent searches for studies published from April 2009 (the publication date of the CMC Classification and Identification criteria) to December 31, 2024 on the correlation between body constitution and NPC (or EBV) by systematically searching English (PubMed, Embase, Web of Science) and Chinese (CNKI, CQVIP, and WanFang) electronic databases. Databases were selected based on the pre‐search and current scholarly fields of CMC. The final search strategy was modified in the discussion section of the three authors after the initial search (File S1).

### 2.2 Eligibility Criteria

This meta‐analysis aimed to synthesize evidence on the distribution of Chinese medicine constitution across the spectrum of health states related to NPC. To this end, we included studies that reported on the following populations: (1) At‐risk individuals: defined as Epstein–Barr virus (EBV)‐positive individuals without a diagnosis of NPC. (2) Patients with NPC (pretreatment): individuals diagnosed with NPC prior to receiving initial treatment. (3) Post‐treatment patients: individuals who had completed initial therapy for NPC.

Studies were included irrespective of language or publication format, provided they reported correlations between CMC distribution and one or more of the above groups.

#### 2.2.1 Inclusion Criteria

(1) Reported data of CMC distribution in one or more of the following NPC‐related groups: at‐risk individuals (EBV‐positive), patients diagnosed with NPC, and post‐treatment patients; (2) CMC Scale based on the *Standard of Classification and Determination of Constitution in TCM* [11] issued by the *China Institute of TCM* published in 2009 and its standard measure [31] for constitution identification in the research; and (3) reporting of clear sample size and complete distribution of constitution types and other demographic information.

#### 2.2.2 Exclusion Criteria

(1) Participants had other systematic serious diseases that may affect the objective; (2) the study focused on the population with a specific type of constitution, such as YiDC; and (3) repeatedly published literature or data.

All authors independently selected studies according to the eligibility criteria set for this review.

### 2.3 Data Extraction and Quality Evaluation

Three authors (Chen, Zi, and Li) worked independently on data extraction and rechecked data before the final integration. The information contained in the included studies consisted of: (1) basic characteristics of each study, including first author, year of publication, time of research period, sample size, and study design; (2) basic information of participants, including period of disease, diagnostic criteria, region, percentage of male participants, and age of the participants; and (3) outcome measures, including the number of patients in each subtype of CMC. The unavailable parts of the data were not used for the final analysis, and the reason for missing data is detailed in the review.

The AHRQ scale recommended by the *Agency for Healthcare Research and Quality* was used in the cross‐sectional study. The Newcastle–Woodward scale (NOS) was chosen for the case–control studies, and the Cochrane collaboration tool was used to evaluate the randomized controlled studies (RCTs). Three major authors independently evaluated the methodology of the identified articles and reached a consensus with another author (Cai).

### 2.4 Data Synthesis and Analysis

The main outcome measures were the single proportion from the cross‐sectional study and the odds ratio (OR) of a case control study with a 95% confidence interval (CI). As TCM constitution and disease status were assessed concurrently, all data were treated as cross‐sectional, and synthesized ORs are interpreted as prevalence odds ratios indicating association, not predictive risk. Data for each constitution type were standardized to enable comparison across studies. A random‐effects model was employed for all meta‐analyses. Between‐study heterogeneity was assessed using the *I* ^2^ statistic (*I* ^2^ ≥ 75% indicating considerable heterogeneity). For meta‐analyses where substantial statistical heterogeneity was present (*I* ^2^ ≥ 75%), pre‐specified subgroup analyses were conducted to explore potential sources of heterogeneity. When only a limited number of studies were available for a comparison marked by substantial clinical heterogeneity (e.g., differing participant health status), statistical pooling was deemed inappropriate, and results were presented descriptively. Sensitivity analyses were performed by serially omitting each study to determine the influence of individual studies on the pooled proportion. Due to the divergent status of the participants, the analysis of CMC type distribution was stratified, analyzed, and presented as forest plots to demonstrate the comparison. Data for the two types of outcome measures were used to detect publication bias, as shown in the funnel plot.

Stata/SE software (version 17.0; Revision 20) was used for data synthesis and illustration. Review Manager 5.4 software was used for quality assessment.

## 3 Results

### 3.1 Study Characteristics

**FIGURE 1:**
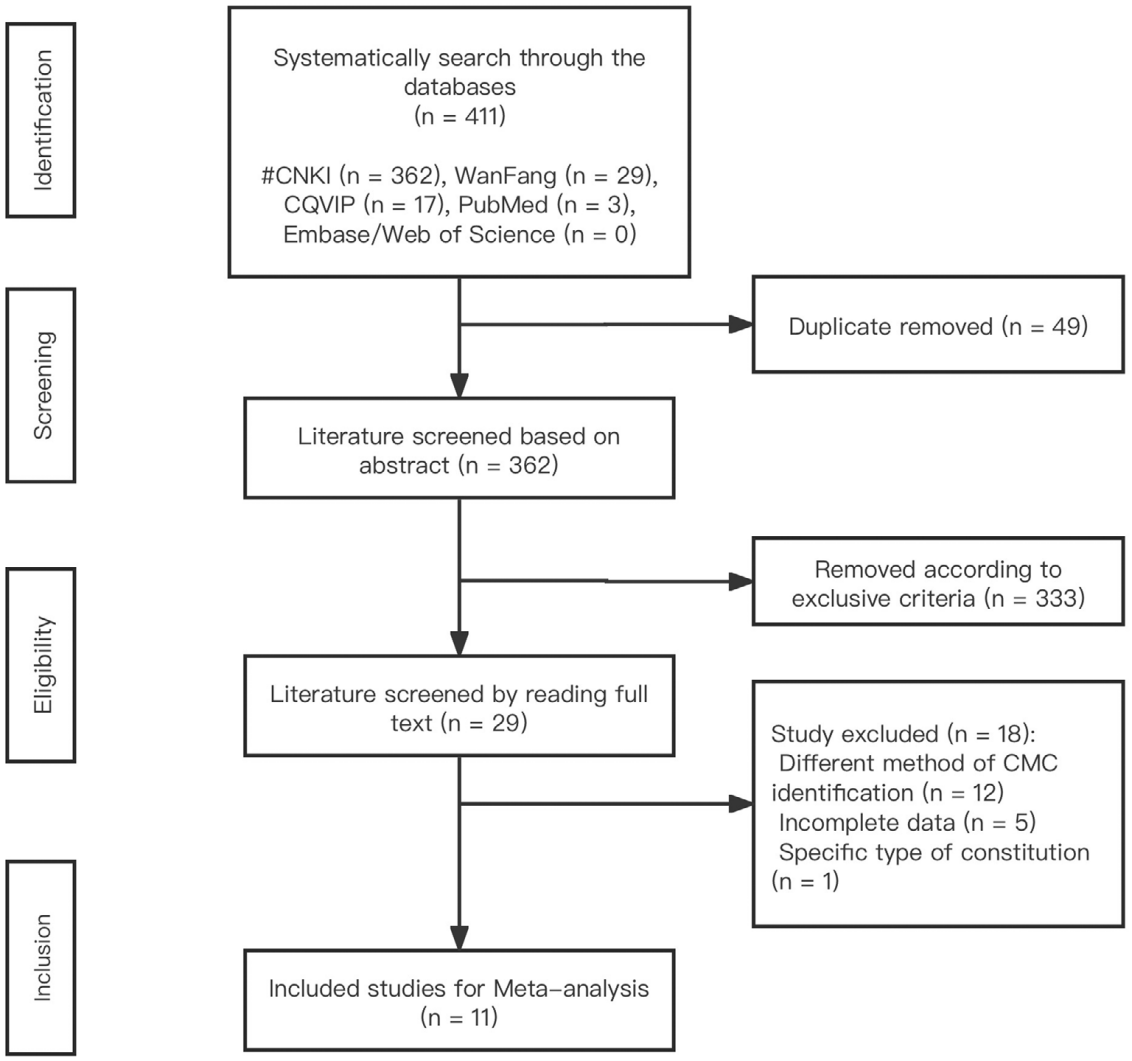
Flow chart of data search and selection.

Eleven studies involving 1174 patients with NPC were included in this review. All studies were conducted in Guangdong, China. Six cross‐sectional studies (*n* = 815), two case–control studies (*n* = 190), and three RCTs (*n* = 169) reporting a correlation between the distribution of constitutions and NPC across related health states were retrieved (Figure 1). The median number of patients per study was 72 (range: 50–309). Five studies involved patients with NPC, of which three focused on patients after treatment and two on NPC‐diagnosed patients, while six other studies recruited EBV‐positive patients. The study characteristics are summarized in Table 1.

**TABLE 1:** Characteristics of included studies.

Study Id	Years	Study design	Research period	Sample size	Participant type	Males (no.%)	Average age (years old)	Region	Inclusive/diagnostic criteria
Liu [32]	2010	CSS	2008.12–2009.3	154	NPC posttreatment 12 months	66.67	Range: (16–68)	Guangzhou, Guangdong, China	Pathological histology diagnosis and accepted radiotherapy treatment with no relapse or metastasis
Chen [33]	2013	CCS	2012.7–2013.2	90 (*n* = 359 for control group)	EBV positive	60.00	Mean (SD): 41.40 (9.68)	Guangzhou, Guangdong, China	VCA‐IgA or NA‐IgA positive
Gao [34]	2014	CCS	2012.1–2012.10	100 (*n* = 100 for control group)	NPC diagnosis	72.81^a^	Mean (SD): 46.35 (13.37)^a^	Guangzhou, Guangdong, China	Pathological histology diagnosis, not receiving treatment
Zhang [35]	2016	CSS	2008.12.1–2014.12.31	309	NPC posttreatment 8–12 weeks	75.08	Mean (SD): 51.53 (12.88)	Zhongshan, Guangdong, China	Patients with NPC accepted comprehensive treatments with no relapse, metastasis or comorbidity
Wang [36]	2018	RCT	2017.1–2018.1	50	EBV positive	42.00	Mean (range): 45.42 (26–68)	Guangzhou, Guangdong, China	VCA‐Ig A and/or EA‐Ig A positive
Li [37]	2019	CSS	2016–2017	72	EBV positive	44.44	Mean (range): 4 4.14 (26–65)	Guangzhou, Guangdong, China	VCA‐Ig A and/or EA‐Ig A positive
Yang [38]	2019	RCT	22017.1–2018.1	56	EBV positive	37.50	Mean (SD, range): 45 (1.50, 26–65)	Guangzhou, Guangdong, China	VCA‐Ig A and/or EA‐Ig A positive
Yang [39]	2019	RCT	2017.1–2018.1	63	EBV positive	41.27	Mean (SD, range): 48.62 (0.29, 26–65)	Guangzhou, Guangdong, China	VCA‐Ig A and/or EA‐Ig A positive
Li [40]	2019	CSS	2018.1–2019.1	61	EBV positive	40.98	Mean (range): 45.55 (22–65)		VCA‐Ig A and/or EA‐Ig A
Lin [41]	2020	CSS	2014.1–2019.12	63	NPC diagnosis	59.00^a^	Mean (SD, range): 48.62 (0.29, 26–65)^a^	Guangzhou, Guangdong, China	Cytological examination or pathological histology diagnosis of malignant tumor with no metastasis
Zhong [42]	2022	CSS	2021.6–2021.11	156	NPC posttreatment 5 years	56.41	U	Guangzhou, Guangdong, China	Pathological histology diagnosis, posttreatment, survival time over 5 years

Abbreviations: CCS, case–control study; CSS, cross‐sectional study; EA‐IgA, Early Antigen Immunoglobulin A; EBV, Epstein–Barr virus; ECA‐IgA, Viral Capsid Antigen Immunoglobulin A; NPC, Nasopharyngeal carcinoma; RCT, random control trails; U, unshown.

^a^ Participant feature of the whole study including our objective population; U, information not provided. Male (no.%): proportion of male participants.

In the quality assessment of the methodology, all six cross‐sectional studies received 6–8 stars of AHRQ as medium‐quality literature, and two case–control studies obtained a score of six on the NOS scale, which is acceptable. The quality of the three RCTs was assessed using the Cochrane risk‐of‐bias evaluation tool. All details are shown in File S2.

### 3.2 Distribution of the Constitution Types Across NPC‐Related Health States

The distribution of BCC ranged from 1.0% (3/309 individuals; 95% CI, 0.3–2.8) for NPC posttreatment at 8–12 weeks to 61.7% (95/154 individuals; 95% CI, 53.8–69.0) for NPC posttreatment at 12 months. Meta‐analytic pooling of the BCC distribution reported by 11 studies yielded an overall proportion of 16.1% (227/1174 individuals; 95% CI, 8.1–24.1), with significant evidence of between‐study heterogeneity (*I* ^2^ = 97.5%, *p* < 0.001).

**FIGURE 2:**
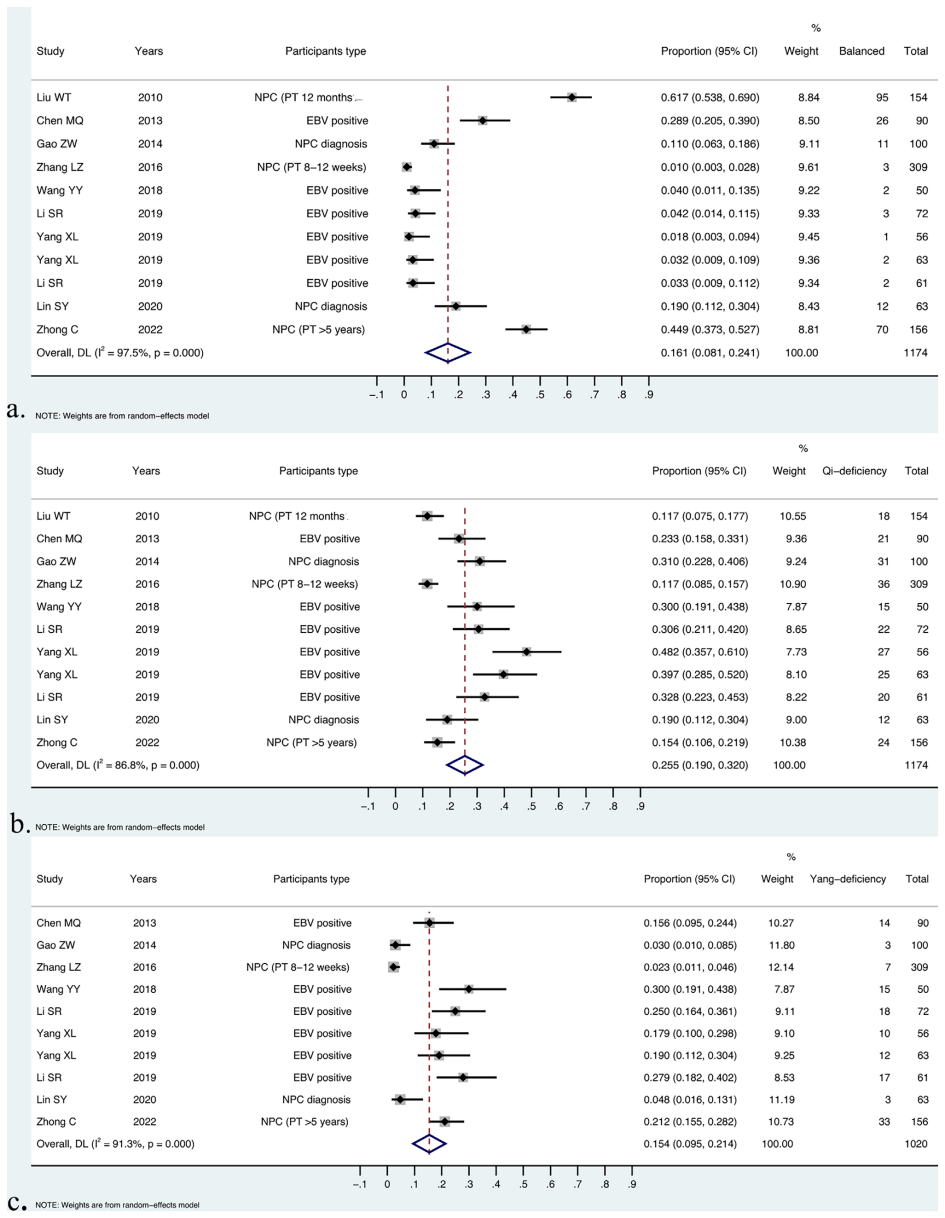
Pooled distribution of BCC, QDC, and YaDC across the related health states of NPC. (a) Pooled BCC proportion across the related health states of NPC; (b) Pooled QDC proportion across the related health states of NPC; (c) Pooled YaDC proportion across the related health states of NPC.

Among the unbalanced constitutions, QDC had a pooled proportion of 25.5% (251/1174 individuals; 95% CI, 19.0–32.0) from 11 studies, with significant evidence of between‐study heterogeneity (*I* ^2^ = 86.8%, *p* < 0.001). The distribution of QDC ranged from 11.7% (18/154 individuals; 95% CI, 7.5–17.7) for posttreatment at 12 months to 48.2% (27/56 individuals; 95% CI, 35.7–61.0) in the EBV‐positive population. Ten studies reported the distribution of YaDC, which ranged from 2.3% (7/309 individuals; 95% CI, 1.1–4.6) for posttreatment at 8–12 weeks to 30.0% (15/50 individuals; 95% CI, 19.1–43.8) in the EBV‐positive population. The pooled distribution of YaDC yielded a result of 15.4% (132/1174 individuals; 95% CI, 9.5–21.4), with significant evidence of between‐study heterogeneity (*I* ^2^ = 91.3%, *p* < 0.001) (Figure 2). The pooled proportions of the YiDC, PDC, DHC, BSC, QSC, and the special inherited constitution (SPC) are listed in Table 2 and Figures S1 and S2 (File S3).

**TABLE 2:** Results of meta‐analysis in each type of constitution.

Proportion (%)	BC	QD	YaD	YiD	PD	DH	BS	QS	SP
Min	1.0	11.7	2.3	2.6	1.0	3.2	0.0	2.0	0.0
Max	61.7	48.2	30.0	28.6	20.1	14.2	12.0	30.0	4.8
Overall	16.1	25.5	15.4	8.5	9.1	8.8	2.6	6.9	2.4
UCI	8.1	19.0	9.5	4.8	5.0	5.8	0.5	3.5	1.0
LCI	24.1	32.0	21.4	12.2	13.1	11.7	4.7	3.5	3.8
*I* ^2^	97.5	86.8	91.3	83.7	86.3	70.7	82.5	85.4	56.0
Posttreatment subgroup	35.7	12.4	3.5	5.5	10.2	10.0	4.1	8.1	2.1
UCI	−6.6	9.8	−7.1	0.4	3.1	0.7	0.4	−0.1	−1.7
LCI	78.1	15.0	29.9	21.0	20.1	13.2	14.4	14.8	4.7
*I* ^2^	99.4	0.0	96.8	95.3	91.8	91.3	89.2	94.4	88.4
EBV‐positive subgroup	6.2	33.4	21.4	5.5	10.2	10.0	0.2	4.1	3.5
UCI	1.5	26.4	16.8	3.2	7.2	7.0	−0.7	2.2	1.7
LCI	11.0	40.4	26.0	7.7	13.2	13.1	1.1	6.1	5.3
*I* ^2^	82.7	56.1	23.5	0.0	0.0	6.4	0.0	0.0	0.0
NPC diagnosis subgroup	14.1	25.2	3.5	16.2	2.1	7.0	8.1	17.1	2.1
UCI	6.4	13.4	0.7	−6.9	−1.3	3.1	1.1	−7.6	−1.3
LCI	21.8	36.9	6.3	39.3	5.4	10.9	15.2	41.8	5.4
*I* ^2^	47.1	67.9	0.0	93.3	42.1	0.0	66.1	95.6	42.1

Abbreviations: BC, balanced constitution; BS, blood‐stasis constitution; DH, dampness‐heat constitution; *I* ^2^, *I*‐squared statistic; LCI, lower confidence interval; PD, phlegm‐dampness constitution; QD, Qi‐deficiency constitution; QS, Qi‐stagnation constitution; SP, special constitution; UCI, upper confidence interval; YaD, Yang‐deficiency constitution; YiD, Yin‐deficiency constitution.

### 3.3 Sensitivity and Subgroup Analyses

Sensitivity analysis was performed to determine the influence of the meta‐analysis of untransformed proportions using the random‐effects inverse‐variance model. The results demonstrated that no individual study significantly affected the overall estimated proportion > 2.3% or over the 95% CI of the proportion (Figures S3–S5; File S3).

**FIGURE 3:**
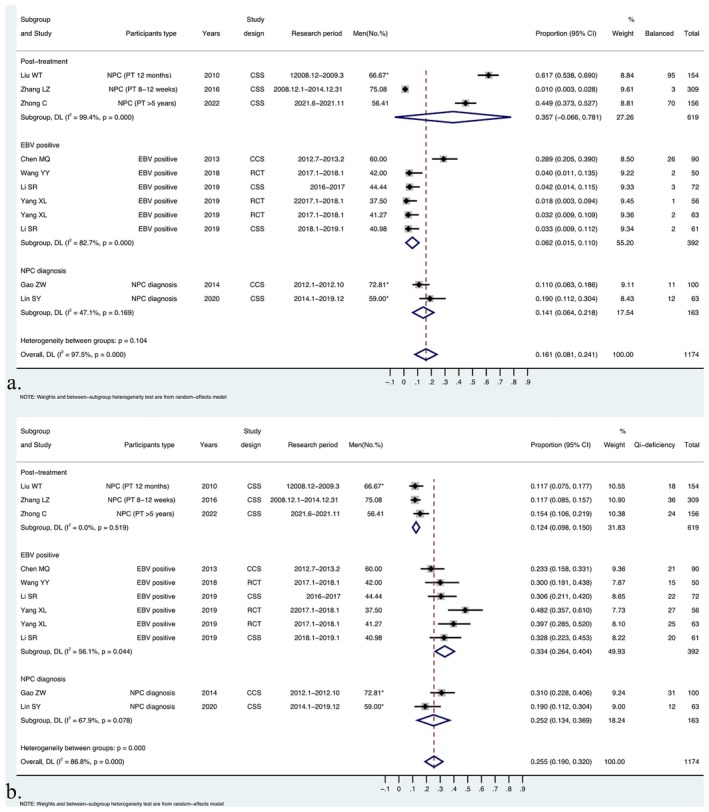
Subgroup analysis of BCC and QDC distribution across the related health states of NPC. (a) Subgroup analysis of the BCC proportion across the related health states of NPC; (b) Subgroup analysis of the QDC proportion across the related health states of NPC.

**FIGURE 4:**
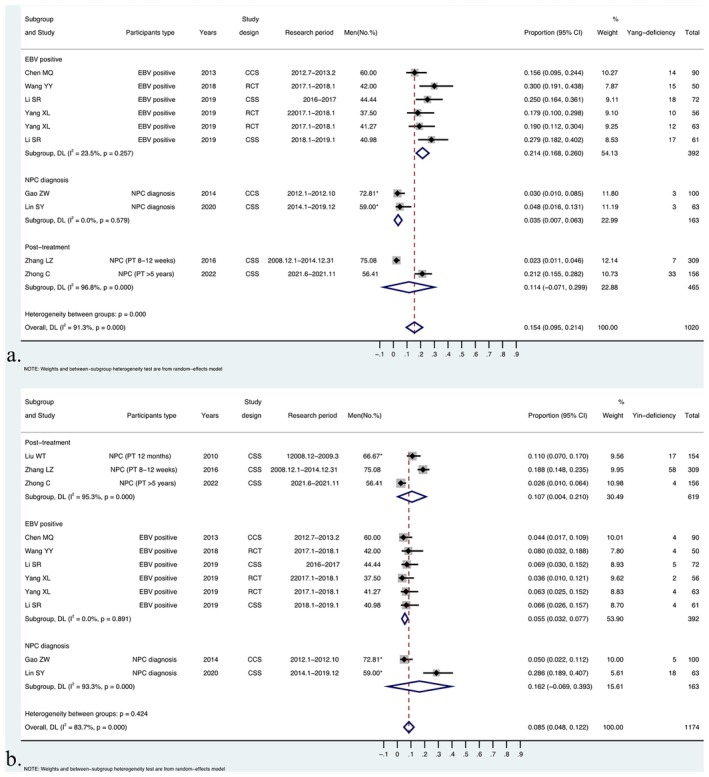
Subgroup analysis of YaDC and YiDC distribution across the related health states of NPC. (a) Subgroup analysis of YaDC proportion across the related health states of NPC; (b) Subgroup analysis of YiDC proportion across the related health states of NPC.

Based on the types of participants, the studies were initially divided into three subgroups: posttreatment, EBV‐positive, and NPC diagnosis groups, and subgroup analysis was performed for all constitutions (Table 2, Figures 3 and 4, and Figures S6–S8; File S3).

In the posttreatment subgroup, considerable heterogeneity was observed in nearly all constitutions, except for QDC, illustrating significant variation among the three different periods of NPC convalescence. Compared with that in the overall proportion, a lower proportion of BCC and YaDC, along with a higher proportion of YiDC, PDC, DHC, BSC, and QSC, were observed in the posttreatment population at 8–12 weeks. Posttreatment patients at 12 months showed a higher distribution of BCC and a lower distribution of QDC and YaDC. In the NPC diagnosis group, considerable heterogeneity was observed in the YiDC and QSC, producing a higher distribution to the pooled distribution of YiDC and QSC in this subgroup. In the EBV‐positive group, a lower heterogeneity was detected for all other types of constitutions apart from BCC. In this subgroup, QDC, YaDC, PDC, and DHC showed higher proportions than BCC.

### 3.4 ORs of the Case–Control Studies

**FIGURE 5:**
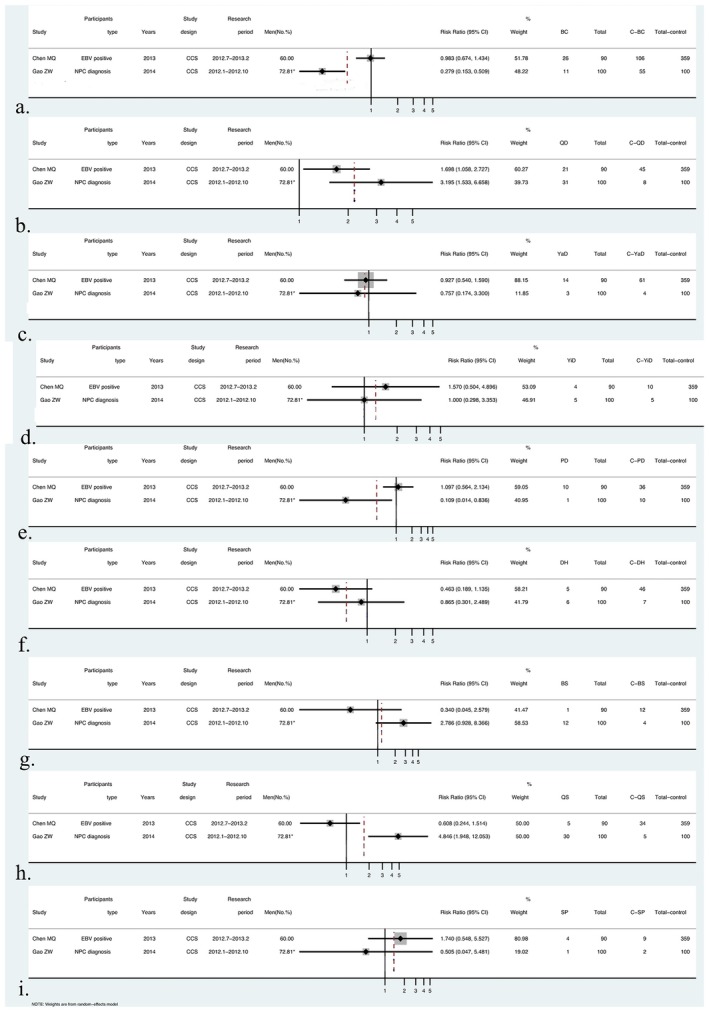
Forrest plot of odds ratio from two case–control studies. Odds ratios for each constitution type are presented separately for the two individual studies of (a) Balanced constitution; (b) Qi‐deficiency constitution; (c) Yang‐deficiency constitution; (d) Yin‐deficiency constitution; (e) Phlegm dampness constitution; (f) Dampness heat constitution; (g) Blood stasis constitution; (h) Qi‐stagnation constitution; (i) Special inherited constitution.

The two available case–control studies, which involved different participant groups (EBV‐positive vs. diagnosed NPC patients), were presented descriptively without statistical pooling due to differing participant health status and outcome definitions (Figure 5).

Based on two cross‐sectional case–control studies, the associations between different constitution types and the two target populations varied. The Qi‐stagnation constitution (QSC) showed the strongest association with diagnosed NPC (OR = 4.846, 95% CI: 1.948–12.053), while the QDC was positively associated with both EBV‐positive individuals (OR = 1.698, 95% CI: 1.058–2.727) and diagnosed NPC patients (OR = 3.195, 95% CI: 1.533–6.658). In contrast, the Balanced constitution and phlegm‐dampness constitution were significantly negatively associated with diagnosed NPC (BCC: OR = 0.279, 95% CI: 0.153–0.509; PDC: OR = 0.109, 95% CI: 0.014–0.850). Although the Yin‐deficiency constitution in EBV‐positive individuals (OR = 1.570, 95% CI: 0.504–4.890) and the Blood‐stasis constitution in diagnosed NPC patients (OR = 2.795, 95% CI: 0.903–8.651) showed positive trends, neither reached statistical significance. The remaining constitutions showed no significant associations in either group.

### 3.5 Assessment of Publication Bias

Publication bias was assessed using the effect size and standard error in the funnel plot based on the proportion of QDC and SPC (Figures S9 and S10; File S3). Visual inspection of the funnel plot revealed asymmetry, which illustrated a lack of studies with a smaller proportion in larger sample sizes, and evidence of small studies' effect (Egger test, *p* = 0.000 for QDC, *p* = 0.018 for SP) was detected.

## 4 Discussion

### 4.1 Summarized Information of the Study

In this review, 11 studies on the association between CMC types and various states across the NPC disease spectrum (including at‐risk individuals, patients with NPC (pre‐treatment), and post‐treatment patients) were meta‐analyzed. QDC was found to be a major type prevalent across the included NPC‐related health states, including EBV‐positive (33.4%) and NPC‐diagnosed (25.2%) subgroups. The definition of QDC is a type of constitution which embodied the daily characteristics of physical functional weakness, especially in the respiratory system, and it was found to be significantly associated with EBV infection status in this review. The overall proportion of YaDC pooled was lower than the distribution of BCC but was 21.4% in the subgroup of the EBV‐positive group. However, in the descriptive comparison of the two case–control studies, YaDC showed a lower prevalence odds ratio for NPC/EBV infection compared with the balanced constitution group. For reference, a study including 2792 healthy physical examinees in the same region (Guangzhou, Guangdong, China) performed in 2008–2009 had reported a distribution of BCC (29.9%), QDC (10.69%), and YaDC (21.24%) [43].

In the subgroup analysis, a high degree of heterogeneity was observed in the posttreatment group, especially at 8–12 weeks. A higher distribution of BCC was found in posttreatment subgroup than in the other subgroups, except for this study of posttreatment at 8–12 weeks, when the highest distribution of YiDC (28.6%), PD (20.1%), and DHC (14.2%) also appeared in this study. Regarding the period after treatment, the side effects of radio chemotherapy could be highly involved in this study, which warrants further research to eliminate the effect of therapy or related syndromes on the constitution.

### 4.2 Limitation and Advantages of This Study

Although the retrieved studies were conducted between 2008 and 2024, the results must be interpreted with caution. This study has a few limitations. The sample size was relatively limited in terms of the number and region. Most of the included studies had a retrospective design or merely used the CMC identification scale as a descriptive tool for patients, which indicated the design of cross sectional in terms of CMC, and was subject to selection bias and considerable heterogeneity. Simultaneously, the subgroup analysis or synthesis of OR was limited by the number of existing studies. These problems may have restricted the extrapolation of the findings of this review.

Despite the limitations, the study has a few strengths. A primary strength is the uniform application of the 2009 TCM constitution standard for methodological comparability. Furthermore, a key strength of this review is its comprehensive analytical perspective. By systematically synthesizing data across the NPC disease spectrum, we move beyond a singular assessment to examining the CMC distributions throughout different states. This approach provides a more holistic understanding of the association between CMC and NPC. Although the outcome measures from other nonunified constitution classifications could not be pooled in this review, these studies were helpful as a foundation for the retrieved studies because they shared the same basis of theoretical origin in TCM [26]. Second, this review sheds light on the confounding factors of this regional disease that affects residents in Asia, which could provide clues for NPC prevention. Furthermore, sensitivity and subgroup analyses were performed on a broader scale of patients with NPC in this review, which could bring a more thorough consideration to different types of constitutions and other periods during the course of NPC for further research [30].

### 4.3 Implication for Further Research

First, the evidence‐based results of this review indicates a consistent association of QDCwith EBV infection and further development of NPC. Apart from the susceptible individuals of NPC, QDC has already been reported as the major type of constitution in many diseases, such as breast and lung cancers in a bibliometric review of 332 clinical studies [44]. The lung and respiratory system play an important role in both TCM and modern medicine theory as a major organ for air‐exchange function, barrier function, and host of complex receptors or sensors for other critical functions [45], and more attention should therefore be paid to the prevention of QDC. Kuang et al. [46] identified 50.6% residents of QDC and more relevant subtypes of constitutions involving QDC in healthy local residents in Guangdong, China, from 2013 to 2018. This investigation indicates the urgency of awareness and action of health administration based on body constitution, especially under subhealthy circumstances and epidemics in public health in recent years [47].

One of the aims of this review was to explore the association between body constitution and NPC morbidity, thereby informing the potential application of CMC in early prevention. In this context, it is noteworthy that some of the included RCTs, although their intervention outcomes were not meta‐analyzed here, have begun to explore the feasibility of modifying constitutions like QDC in at‐risk populations (e.g., EBV‐positive individuals) [36]. In this review, the distribution of YaDC was also noteworthy in EBV‐positive patients but was not indicated to be a risk factor compared with the normal group. This finding may provide a new view of the initiation of disease from an EBV‐positive status to NPC morbidity and the regional effect on the constitution of healthy individuals. The proportion of PDC and DHC were not notably elevated in the participants assessed in this review but may be related to the side effects of the therapy. As a confounding factor, the distribution of DHC was also predominant in local healthy residents compared with those in the northern city of China (Beijing), according to another CMC study [48].

However, further studies are required to confirm these findings. More unsolved problems in constitutional studies were revealed in the writing process of this review. First, the specific enrollment in Guangzhou in the current research requires more cases from other high‐incidence regions, such as Guangxi and Jiangxi in China or Malaysia in Southeast Asia [6, 49]. Second, methodology of CMC identification standardization and the quality assessment of relative studies needs more attention and improvement. Third, the study of CMC still needs to address the identification of constitution from symptoms, syndromes, or side effects caused by treatment, so that the persistent constitution or the long‐term effects reflected in the change of constitution can be fully clarified.

In this review, we chose to attach importance to the domestic residents' body constitution, which concerns the incidence of viral diseases and even more severe situations, such as cancer. We also suggest the application of CMC identification in the prevention or recovery of NPC through constitution‐based intervention/treatment. As suggested by RCTs of EBV‐positive patients and CMC intervention, this has started to answer the critical question in CMC of “could the constitution be improved,” and more high‐quality studies and prospective research are expected in the future to determine if improving specific unbalanced constitutions (e.g., QDC) before illness onset can effectively reduce the risk of NPC.

## 5 Conclusion

This systematic review synthesized evidence from 11 studies on CMC across health states related to NPC. The Qi‐deficiency constitution (QDC) was the most prevalent type (25.5%) and was associated with an increased risk of NPC/EBV infection. The proportion of Yang‐deficiency constitution (YaDC, 15.4%) was also notable, particularly in the EBV‐positive subgroup (21.4%). Given the multifactorial etiology of NPC, assessing TCM constitution offers a holistic perspective for identifying susceptibility in at‐risk individuals and informing preventive strategies. Further high‐quality prospective studies are needed to strengthen the evidence base for public health and clinical application.

## Author Contributions

**Shun‐Qi Chen:** conceptualization, data curation, formal analysis, investigation, methodology, software, supervision, visualization, writing – original draft. **Yan Zi:** data curation, formal analysis, investigation, methodology. **Wen‐Le Li:** data curation, investigation, methodology. **Yu‐Yang Cai:** formal analysis, investigation, visualization. **Geng‐Shuo Miao:** formal analysis. **Huai‐Yu Wang:** writing – review and editing. **Ming‐Hua Bai:** writing – review and editing. **Ji Wang:** funding acquisition, methodology, project administration, supervision, validation, writing – review and editing.

## Conflicts of Interest

The authors declare no conflicts of interest.

## Supporting information


**File S1:** cam471641‐sup‐0001‐supinfoS1.docx.

**File S2:** cam471641‐sup‐0002‐supinfoS2.pdf.

**File S3:** cam471641‐sup‐0003‐supinfoS3.docx.

## Data Availability

Data available on request from the authors.
