# Supplementary material for: Association Between Nasopharyngeal Carcinoma and Chinese Medicine Constitution: A Meta‐Analysis
Source: Cancer Med. 2026 Mar 26;15(4):e71641. doi: 10.1002/cam4.71641 (PMC13140684; doi:10.1002/cam4.71641)
Supplement: Supplementary file 2 — File S2: cam471641‐sup‐0002‐supinfoS2.pdf. [file CAM4-15-e71641-s003.pdf]

Supplement File 2. Quality Evaluation

Table 1.AHRQ scale for cross-sectional study quaility assessment

AHRQ		Zhang LZ, 2016	Li SR, 2019	Liu WT, 2010	Li SR, 2019.6	Lin SY, 2016	Zhong C, 2022
1	1)Define the source of information(survey,record review)	1	1	1	1	1	1
2	2)List inclusion and exclusion criteria for exposed and unexposed subjects(cases and controls)or refer to previous publications	1	1	1	1	1	1
3	a2)List inclusion and exclusion criteria for exposed and unexposed subjects (eases and controls)or refer to previous publications	1	1	1	1	1	1
4	4)Indicate whether or not subjects were consecutive if not population-based	0	0	0	0	1	0
5	5)Indicate if evaluators of subjective components of study were masked to other aspects of the status of the participants	0	0	0	0	0	0
6	6)Describe any assessments undertaken for quality assurance purposes(e.g.test/retest of primary outcome measurements)	1	1	1	1	1	1
7	7)Explain any patient exclusions from analysis	1	1	1	1	1	1
8	8)Describe how confounding was assessed and/or controlled.	1 (	0	1	1	0	0
9	9)If applicable,explain how missing data were handled in the analysis	1	1	0	0	1	1

10	**10)Summarize patient response rates and completeness of data collection**	0	0	0	0	1	0
11	**11)Clarify what follow-up,if any,was expected and the percentage of patients for which incomplete data or follow-up was obtained**	0	0	0	0	0	1
**Total score**		7	6	6	6	8	7

Table 2.Newcastle-Ottawa Quality Assessment Scale-Case Control Studies

NOS (A maximum of one star for each item in Selection and Exposure catagories, two stars in Comparability)		Chen MQ, 2013	Gao ZW, 2014
Selction	1)Is the case definition adequate?<br>a)yes,with independent validation*<br>b)yes,eg record linkage or based on self reports<br>c)no description	1	1
	2)Representativeness of the cases<br>a)consecutive or obviously representative series of cases*<br>b)potential for selection biases or not stated	0	0
	3)Selection of Controls<br>a)community controls*<br>b)hospital controls<br>c)no description	1	1
	4)Definition of Controls<br>a)no history of disease (endpoint)*<br>b)no description of source	1	1
Conparabli ty	1)Comparability of cases and controls on the basis of the design or analysis<br>a)study controls for______;(Select the most important factor.)*	0	0
	b)study controls for any additional factor *<br>(This criteria could be modified to indicate specific	1	1

	control for a second important factor.)		
Exposure	1)Ascertainment of exposure<br>a)secure record(eg surgical records)*<br>b)structured interview where blind to case/control status*<br>c)interview not blinded to case/control status<br>d)written self report or medical record only<br>e)no description	0	0
	2)Same method of ascertainment for cases and controls<br>a)yes*<br>b)no	1	1
	3)Non-Response rate<br>a)same rate for both groups*<br>b)non respondents described<br>c)rate different and no designation	1	1
Total Score(stars)		6	

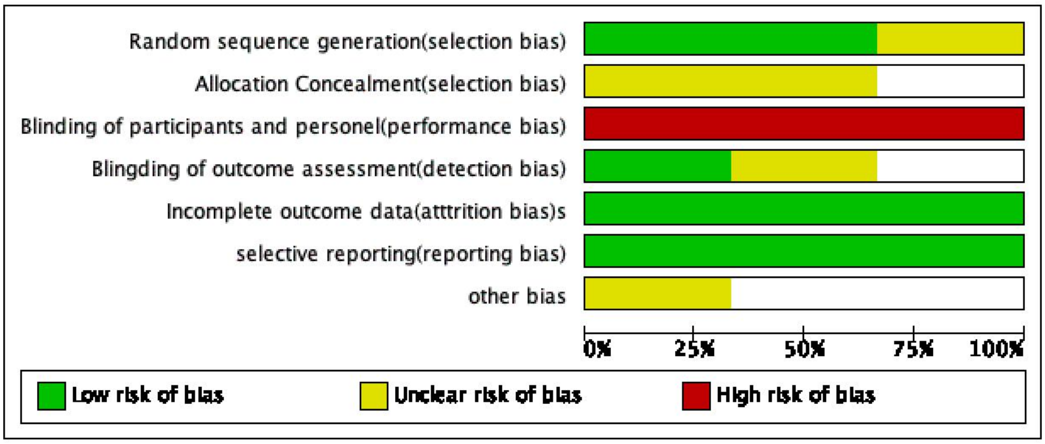

Figure 1.Methodlogy Quality Assessment of 3 RCTs
