# Supplementary material for: Association Between Nasopharyngeal Carcinoma and Chinese Medicine Constitution: A Meta‐Analysis
Source: Cancer Med. 2026 Mar 26;15(4):e71641. doi: 10.1002/cam4.71641 (PMC13140684; doi:10.1002/cam4.71641)
Supplement: Supplementary file 3 — File S3: cam471641‐sup‐0003‐supinfoS3.docx. [file CAM4-15-e71641-s002.docx]

**Supplement File 3. eFigures and eTables**

| **Abreviation of constitution** | **Full Name** |
| --- | --- |
| BCC | Balanced Constitution |
| BSC | Blood-Stasis Constitution |
| DHC | Dampness-Heat Constitution |
| PDC | Phlegm-Dampness Constitution |
| QDC | Qi-Deficiency Constitution |
| QSC | Qi-Stagnation Constitution |
| SPC | Special Inherited Constitution |
| YaDC | Yang-Deficiency Constitution |
| YiDC | Yin-Deficiency Constitution |


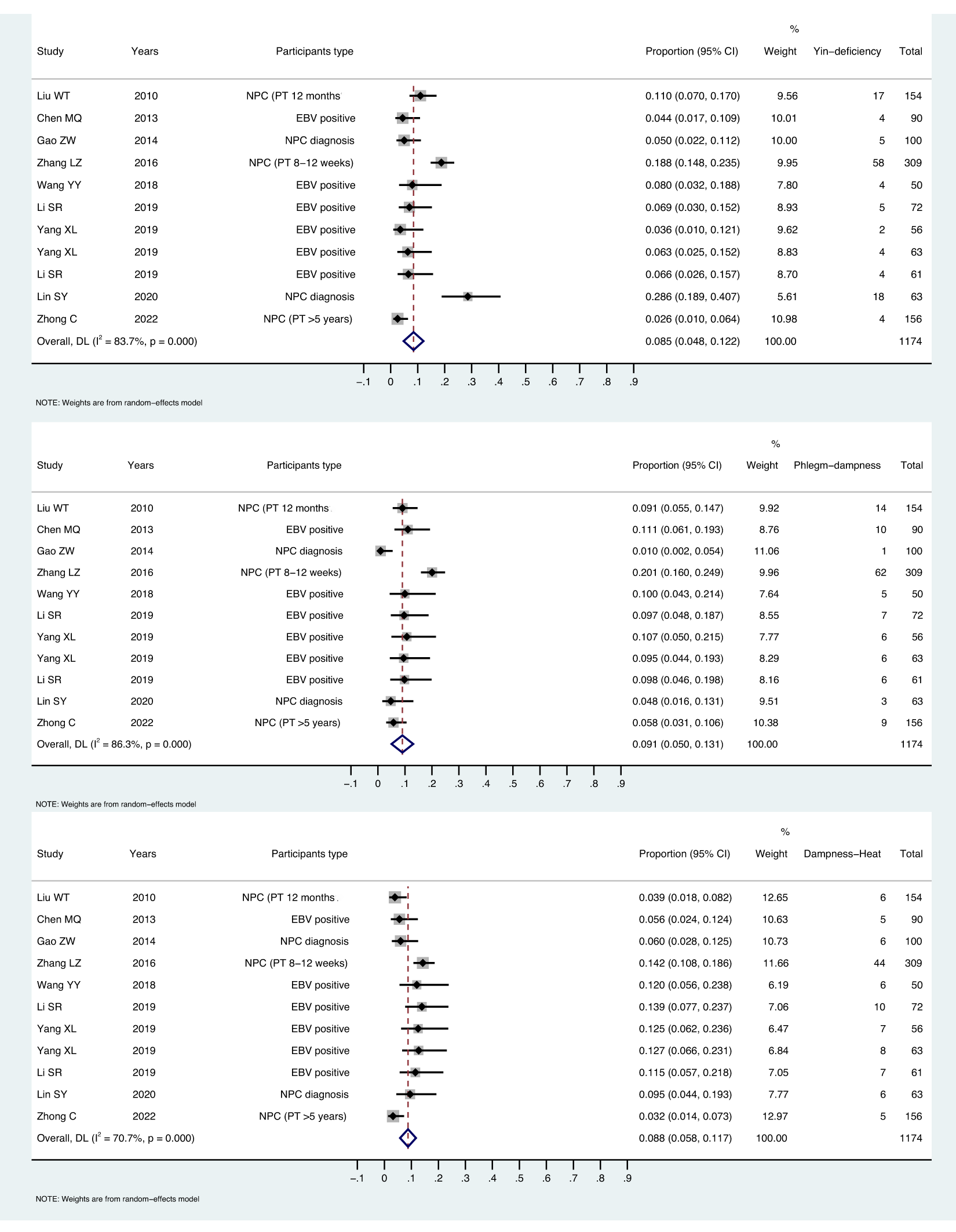


**Figure S1. Overall distribution of YiDC, PDC and DHC across the related health states of NPC**

**
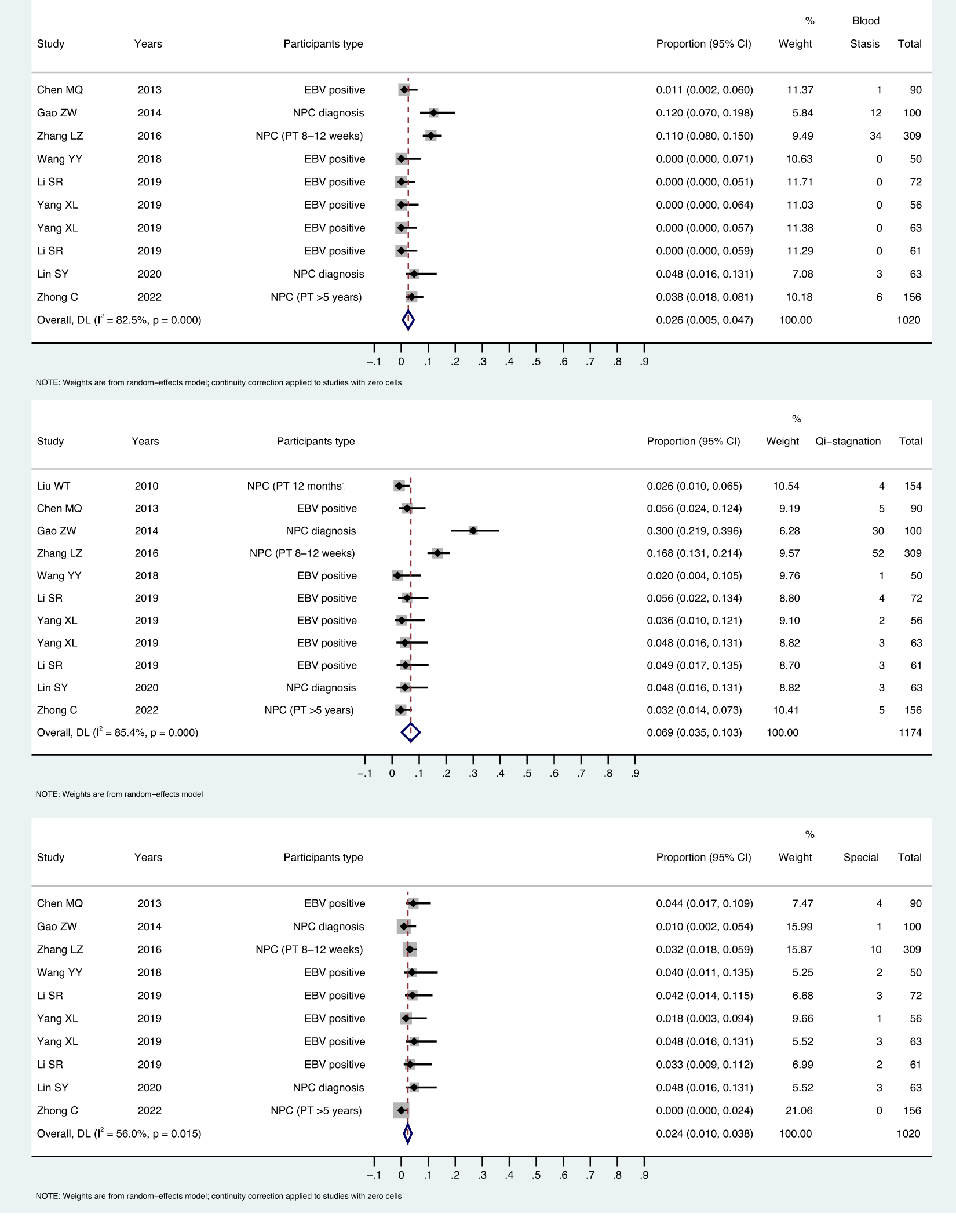
**

**Figure S2. Overall distribution of BSC, QSC and SPC across the related health states of NPC**

**
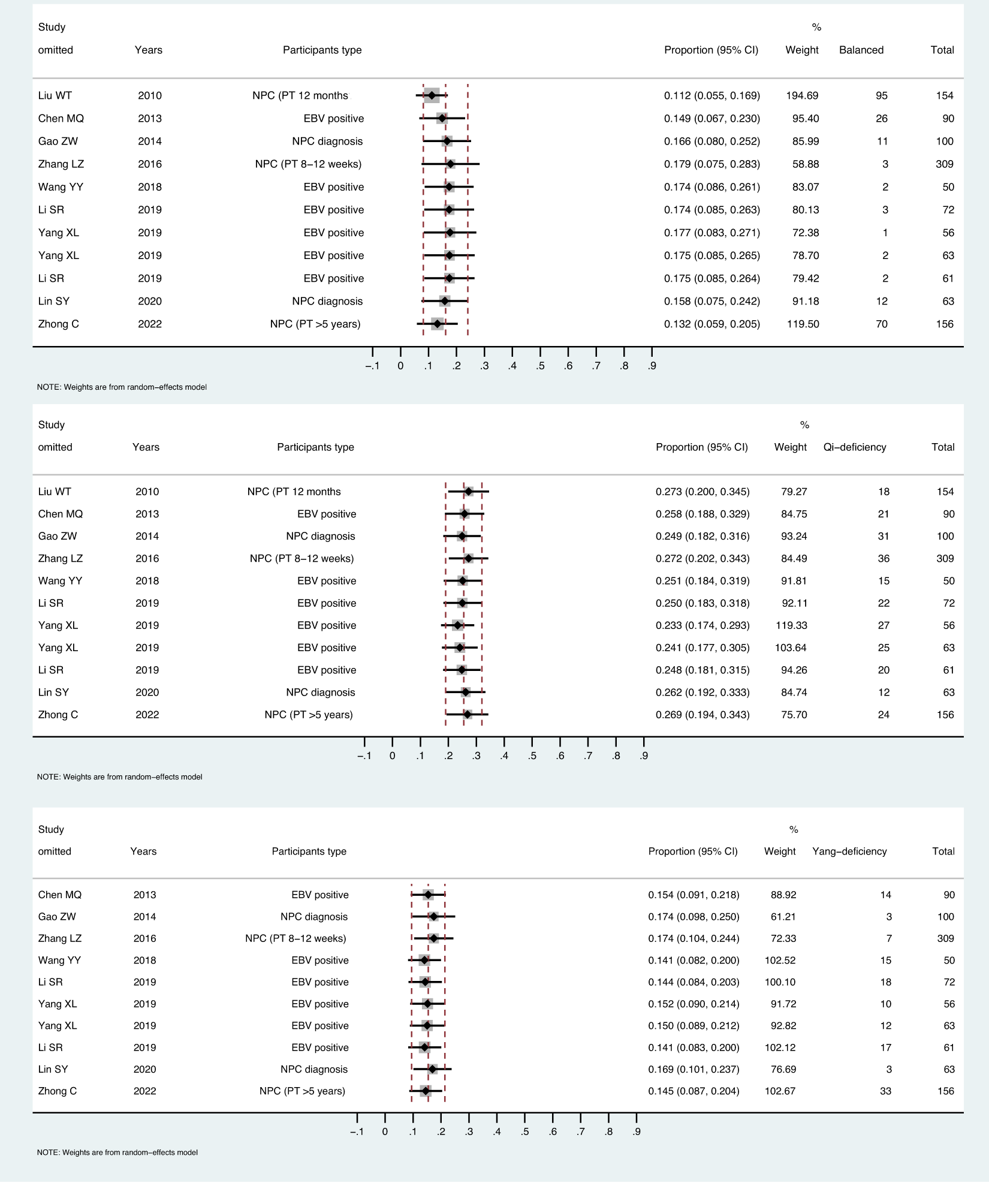
**

**Figure S3. Sensitivity analysis of the influence from each article in distribution of BCC, QDC and YaDC**

**
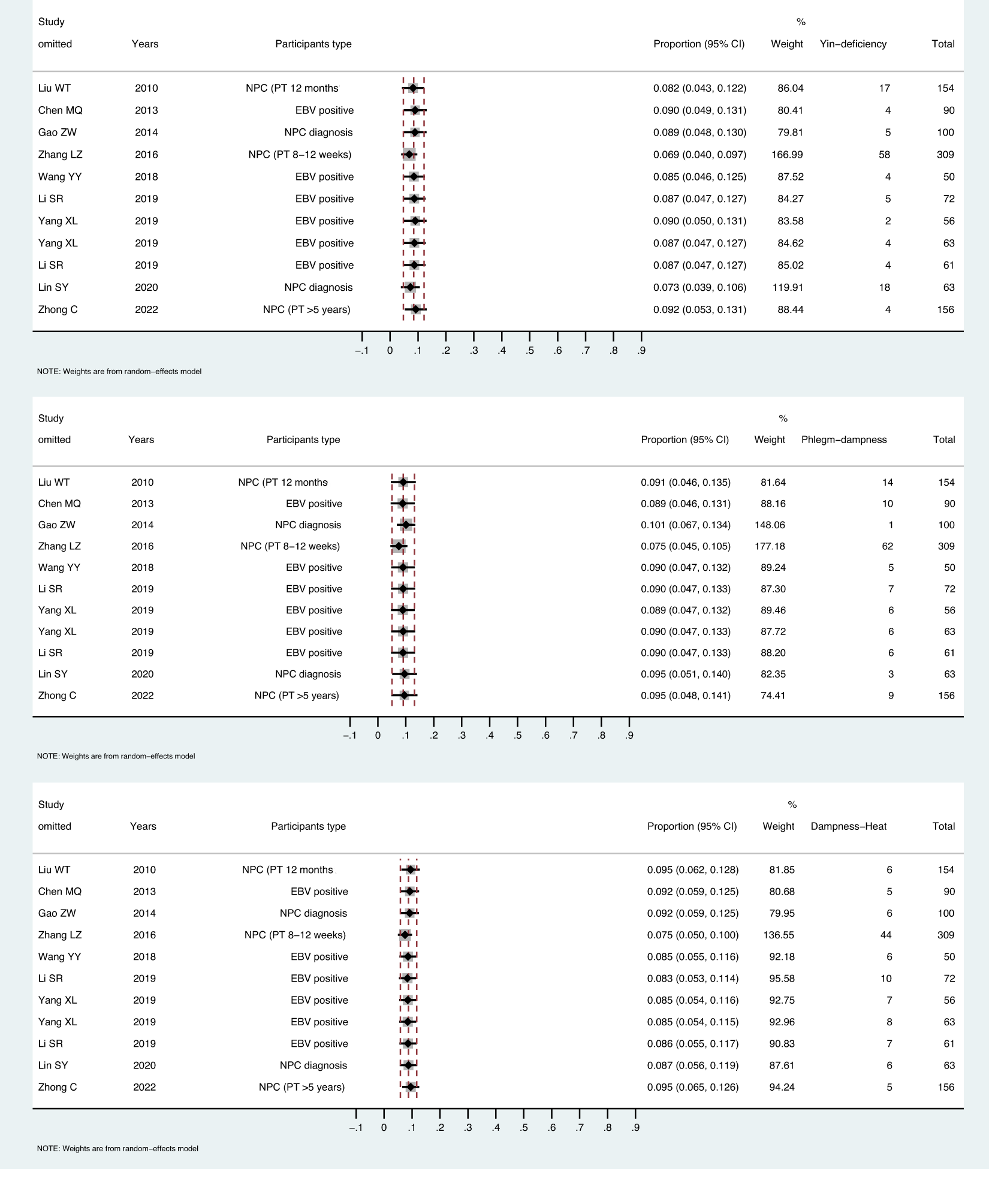
**

**Figure S4. Sensitivity analysis of the influence from each article in distribution of YiDC, PDC, DHC**

**
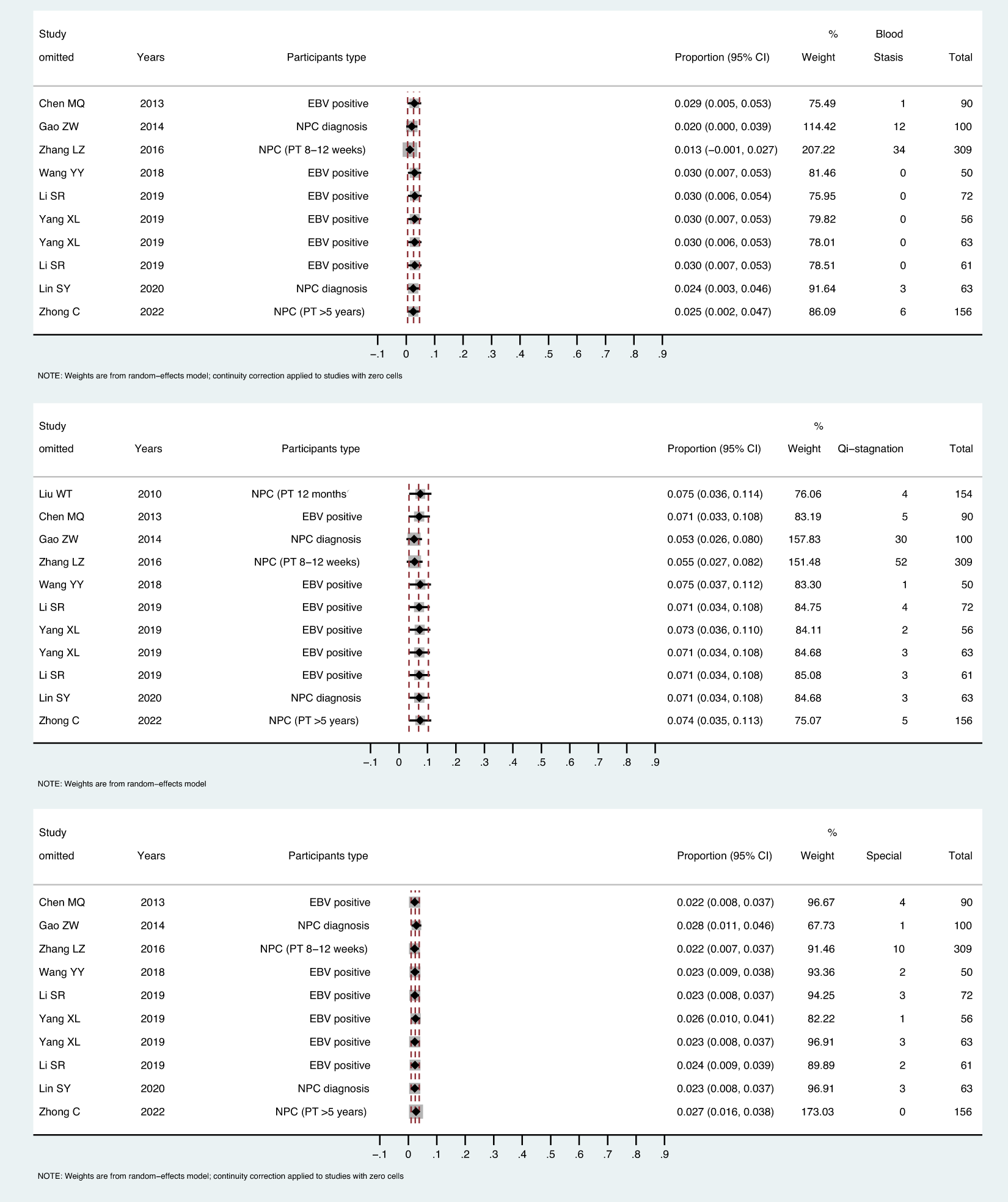
**

**Figure S5. Sensitivity analysis of the influence from each article in distribution of BSC, QSC and SPC**

**
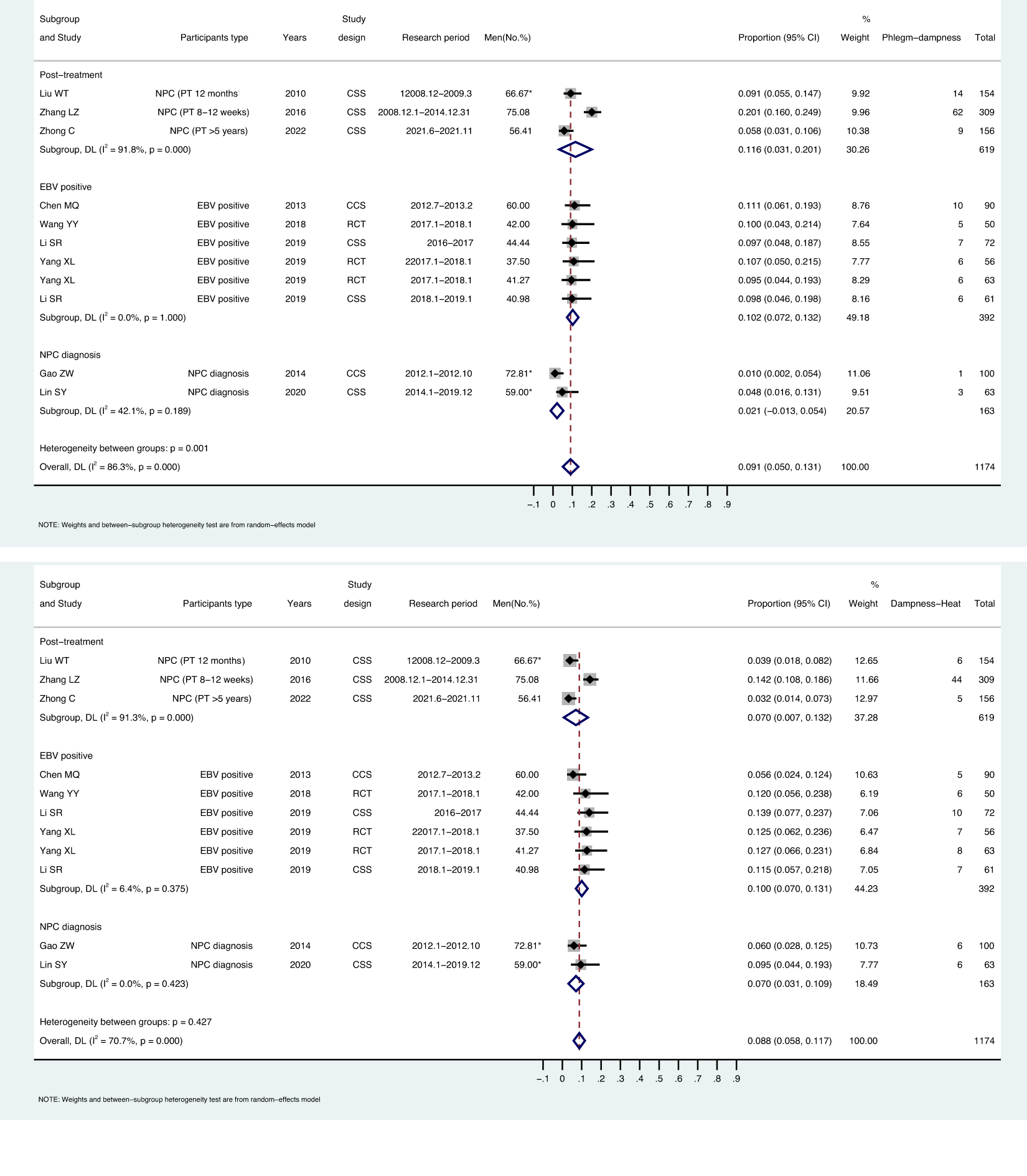
**

**Figure S6. Subgroup analysis of PDC and DHC distribution across the related health states of NPC**

**
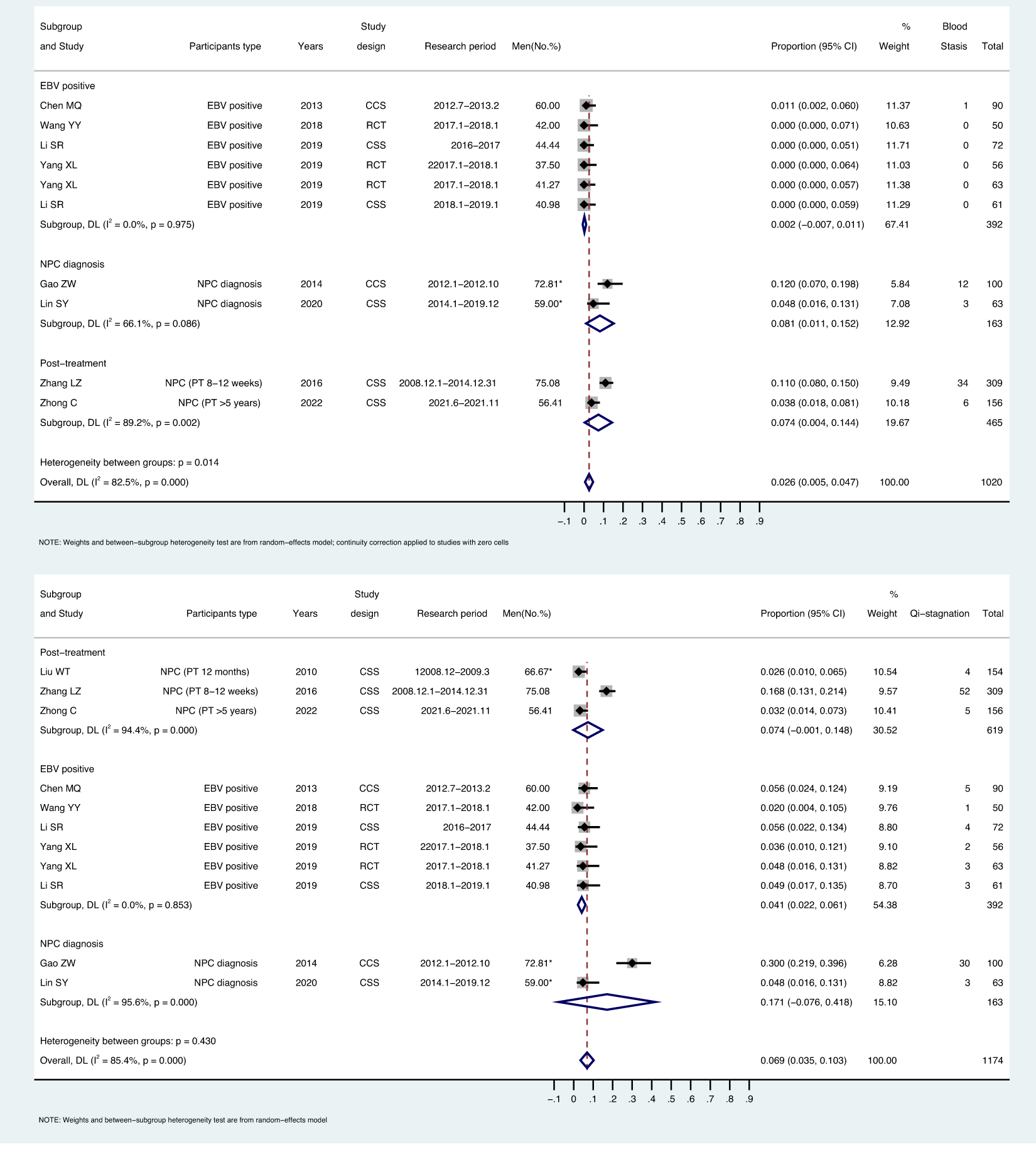
**

**Figure S7. Subgroup analysis of BSC and QSC distribution across the related health states of NPC**

**
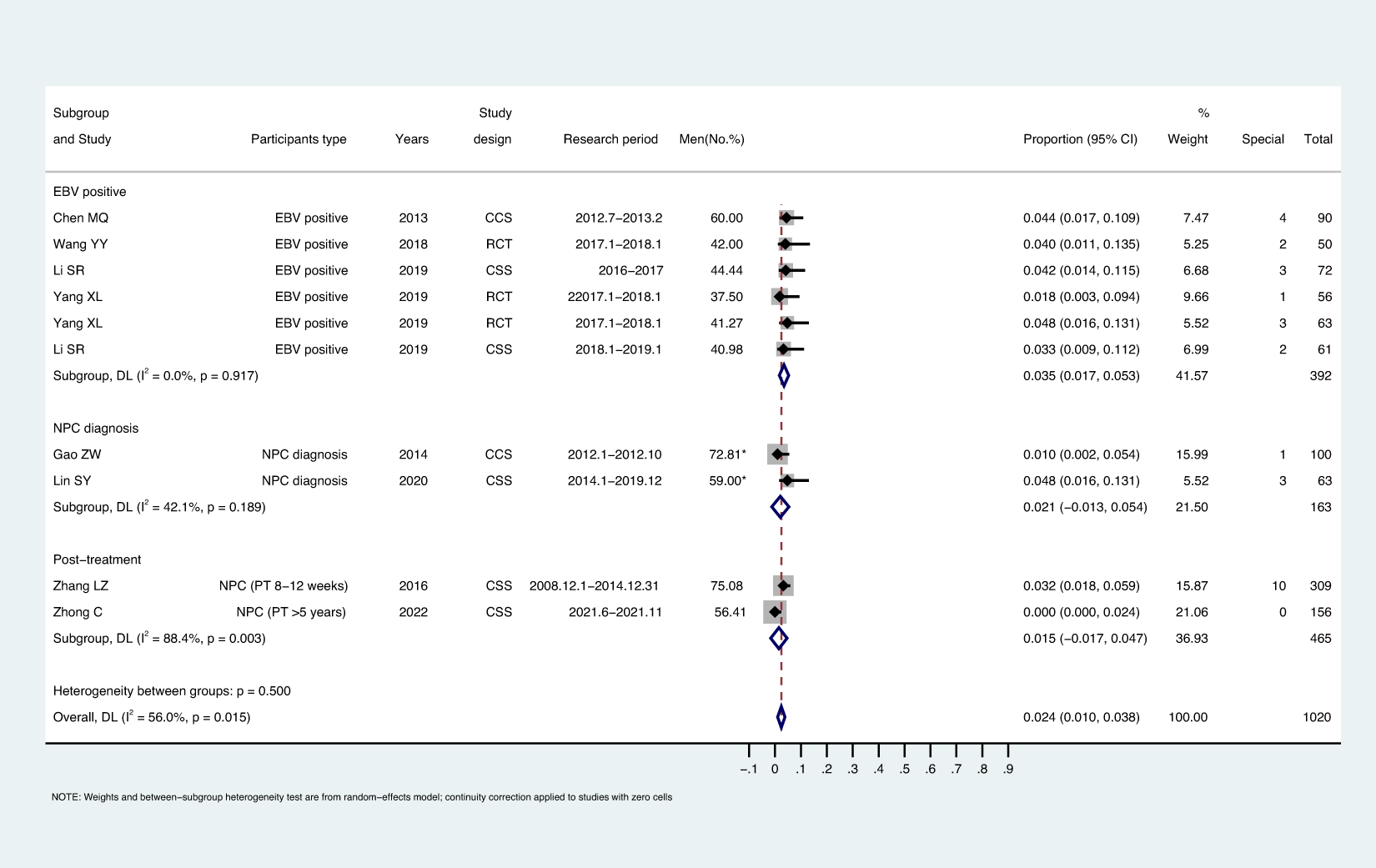
**

**Figure S8. Subgroup analysis of SPC distribution across the related health states of NPC**

**
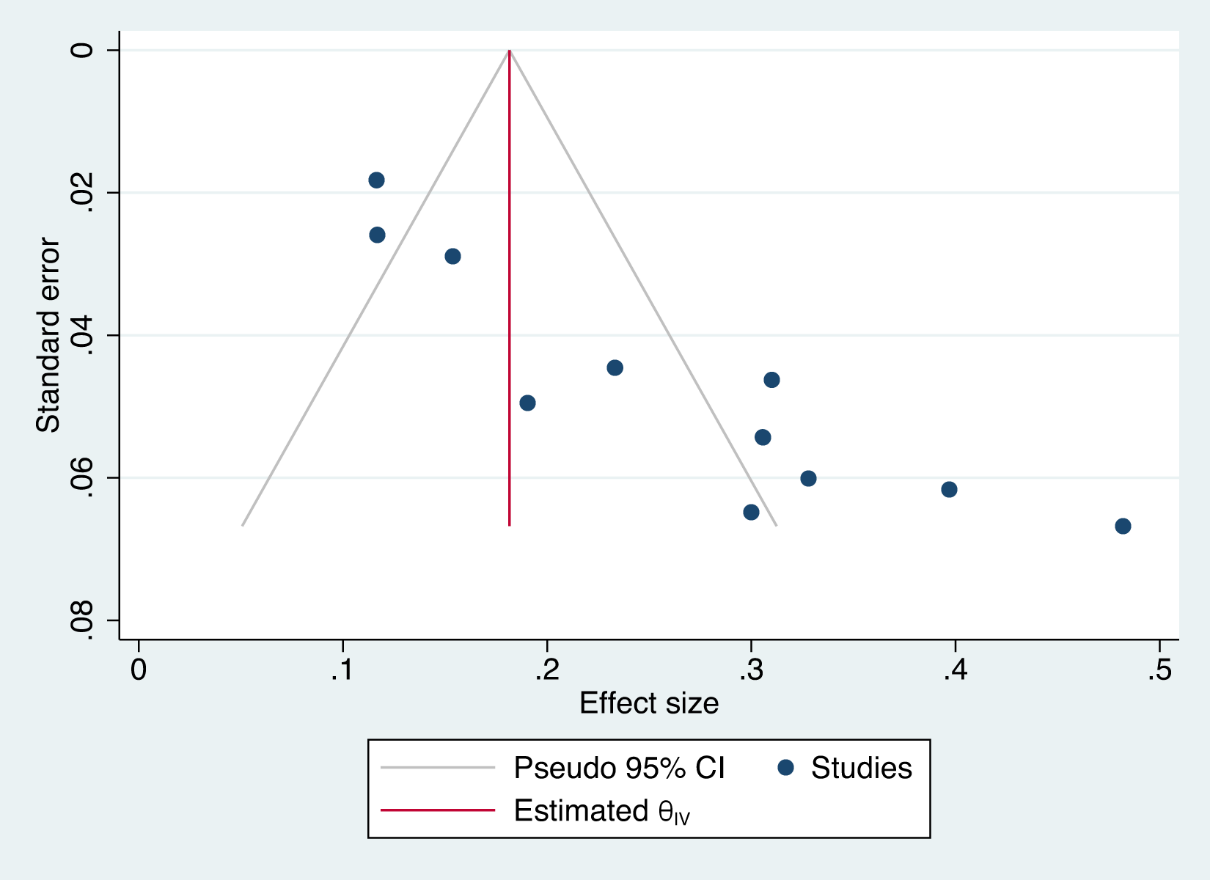
**

**Figure S9. Funnel plot of the distribution of Qi-deficiency constitution** (**Egger test, P =0.000)**

**
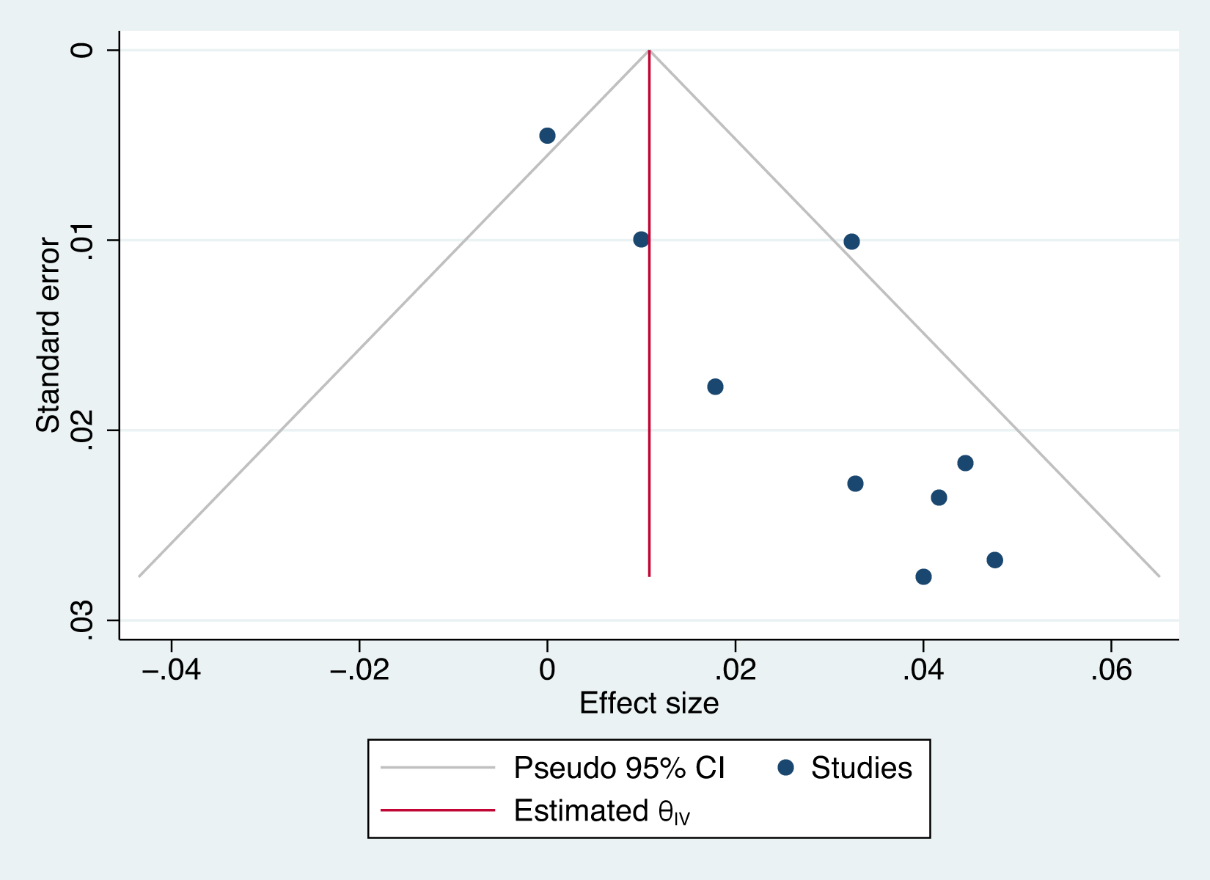
**

**Figure S10. Funnel plot of the distribution of Special constitution (Egger test, P=0.018)**
